# Metagenomics analysis of sewage for surveillance of antimicrobial resistance in South Africa

**DOI:** 10.1371/journal.pone.0309409

**Published:** 2024-08-26

**Authors:** Anthony M. Smith, Masindi Ramudzulu, Patrick Munk, Baptiste J. P. Avot, Kerneels C. M. Esterhuyse, Nico van Blerk, Stanford Kwenda, Phuti Sekwadi

**Affiliations:** 1 Division of the National Health Laboratory Service, National Institute for Communicable Diseases, Centre for Enteric Diseases, Johannesburg, South Africa; 2 Faculty of Health Sciences, Department of Medical Microbiology, School of Medicine, University of Pretoria, Pretoria, South Africa; 3 National Food Institute, Technical University of Denmark, Copenhagen, Denmark; 4 Waste Water Treatment, Tshwane, South Africa; 5 Ekurhuleni Water Care Company, Kempton Park, South Africa; North Carolina State University, UNITED STATES OF AMERICA

## Abstract

Our 24-month study used metagenomics to investigate antimicrobial resistance (AMR) abundance in raw sewage from wastewater treatment works (WWTWs) in two municipalities in Gauteng Province, South Africa. At the AMR class level, data showed similar trends at all WWTWs, showing that aminoglycoside, beta-lactam, sulfonamide and tetracycline resistance was most abundant. AMR abundance differences were shown between municipalities, where Tshwane Metropolitan Municipality (TMM) WWTWs showed overall higher abundance of AMR compared to Ekurhuleni Metropolitan Municipality (EMM) WWTWs. Also, within each municipality, there were differing trends in AMR abundance. Notably, within TMM, certain AMR classes (macrolides and macrolides_streptogramin B) were in higher abundance at a WWTW serving an urban high-income area, while other AMR classes (aminoglycosides) were in higher abundance at a WWTW serving a semi-urban low income area. At the AMR gene level, all WWTWs samples showed the most abundance for the *sul1* gene (encoding sulfonamide resistance). Following this, the next 14 most abundant genes encoded resistance to sulfonamides, aminoglycosides, macrolides, tetracyclines and beta-lactams. Notably, within TMM, some macrolide-encoding resistance genes (*mefC*, *msrE*, *mphG* and *mphE*) were in highest abundance at a WWTW serving an urban high-income area; while *sul1*, *sul2* and *tetC* genes were in highest abundance at a WWTW serving a semi-urban low income area. Differential abundance analysis of AMR genes at WWTWs, following stratification of data by season, showed some notable variance in six AMR genes, of which *bla*_*KPC-2*_ and *bla*_*KPC-34*_ genes showed the highest prevalence of seasonal abundance differences when comparing data within a WWTW. The general trend was to see higher abundances of AMR genes in colder seasons, when comparing seasonal data within a WWTW. Our study investigated wastewater samples in only one province of South Africa, from WWTWs located within close proximity to one another. We would require a more widespread investigation at WWTWs distributed across all regions/provinces of South Africa, in order to describe a more comprehensive profile of AMR abundance across the country.

## Introduction

Antimicrobial resistance (AMR) is a major global healthcare problem, which affects all countries regardless of income levels [1–3]. AMR has been recognized as a ‘One Health’ problem [4]. The World Health Organization (WHO) has declared that AMR is one of the top ten global public health threats facing humanity [5]. Therefore, it is important to have good surveillance systems in place to monitor the global prevalence and spread of AMR. In 2015, the WHO launched the Global Antimicrobial Resistance Surveillance System (GLASS) to support its global action plan on AMR [6]. GLASS encourages countries to enroll into its program and move to surveillance approaches based on systems that include data at multiple levels including laboratory, epidemiological, clinical, and population-level data. However, most current AMR surveillance systems are still focused on a limited number of human pathogens (isolate-based surveillance) and mainly associated with hospitalized patients [7]. This narrow pathogen spectrum therefore does not capture all relevant AMR genes. As such, a major proportion of AMR genes actually goes undetected in the commensal bacterial flora of healthy individuals.

An innovative adjunct to current conventional methodologies for surveillance of AMR in the human population is that of analysis of raw (untreated) sewage [8]. For surveillance activities in the human population, raw sewage is a very appealing specimen to work on. Wastewater-based epidemiology can provide insight into the upstream human population, especially if the municipal wastewater treatment works (WWTW) mainly receives domestic wastewater without significant contributions from other sources like farming and agriculture. Also, there are no ethical concerns associated with analysis of sewage, as data cannot be linked to any individual. Raw sewage is already well-described as a good specimen on which pathogen surveillance activities can be conducted. These include surveillance for polio [9–11] and more recently for SARS-CoV-2 [12,13]. However, most of these types of sewage surveillance activities target a single or limited set of pathogens. The more comprehensive approach to raw sewage analysis for investigation of both pathogen and AMR presence (and abundance) is a metagenomics analysis approach. Metagenomics is a culture-independent methodology that involves the direct sequencing of mixed genomic (genetic) material present in a sample [14].

Over recent years, the application of metagenomics analysis of raw sewage for investigation of AMR, has gained traction, with numerous publications describing use of the technology [15–20]. Hendriksen *et al* [15] performed a global analysis of sewage from 79 sites in 60 countries; to show that systematic differences in abundance and diversity of AMR genes exist in Europe/North-America/Oceania versus Africa/Asia/South-America. Petersen *et al* [17] performed an analysis of toilet waste from 18 international airplanes arriving in Copenhagen International Airport from nine cities in three world regions; to show that a higher abundance and diversity of AMR genes are carried on airplanes from South Asia as compared to North America. In low- and middle-income countries, there is a lack of wastewater metagenome data which prevents comparable surveillance studies. Particularly, very few African laboratories have applied metagenomics sequencing to investigate raw sewage and wastewater, thus, published sewage metagenomic data from Africa is limited. Some publications out of Africa only report on the use of metagenomics data to describe the prevalence of microbial populations [21], while other publications have extended on analysis of metagenomics data to describe the prevalence and abundance of AMR [22–25]. Overall, in Africa very little is known about the presence, diversity and abundance of AMR in raw sewage. Martiny *et al* [26] reported limited metagenomics data coming out of African countries; they investigated all metagenomics data in the public repositories of genomic data to find that the sampling location of data is massively skewed towards European and North American countries.

In this study, we describe a 2-year longitudinal study which included a metagenomics analysis of raw untreated sewage received at four WWTW in the Gauteng Province of South Africa—these included two WWTW servicing semi-urban low-income areas and two WWTWs servicing urban high income areas. The study period ran for 24 months from December 2020 to November 2022, and included monthly sewage sampling and metagenomic analyses of the resistome. To the best of our knowledge, this is the first study of its kind from South Africa.

## Materials and methods

### Study setting

This study was set in two metropolitan municipalities (Ekurhuleni and Tshwane) located in the Gauteng Province of South Africa (https://www.arcgis.com/home/item.html?id=1976f2e85e43497b8cc2b14ab7a8400c), to include a total of four WWTWs. Written permissions were obtained from municipalities in order for us to conduct this study. Service level agreements (including all permissions) were signed with the Ekurhuleni Water Care Company, Ekurhuleni Metropolitan Municipality (EMM) and the Utility Services Department, Tshwane Metropolitan Municipality (TMM). The two metropolitan municipalities were within close proximity (~60 km) to one another. The EMM included a WWTW which serves a semi-urban low-income area (named, EMM semi-urban) and a WWTW which serves an urban high-income area (named, EMM urban). The TMM included a WWTW which serves a semi-urban low-income area (named, TMM semi-urban) and a WWTW which serves an urban high income area (named, TMM urban). Table 1 summarizes details of the four selected WWTW, the geographical area served by the WWTW and metadata for the population living within these areas.

**Table 1:** Summary of metadata for the geographical area and human population served by the WWTWs.

**Name of WWTW**	TMM semi-urban	TMM urban	EMM semi-urban	EMM urban
**Location of WWTW (Province, Municipality)**	Gauteng Province, Tshwane Municipality	Gauteng Province, Tshwane Municipality	Gauteng Province, Ekurhuleni Municipality	Gauteng Province, Ekurhuleni Municipality
**Areas serviced by the WWTW**	Soshanguve, Mabopane	Waverley, Brummeria, Silverton, Hillcrest, Colbyn, Hatfield, Waterkloof, Menlo Park	Tsakane	Farramere, Alphen Park, Lakefield, Westdene, Northmead, Rynfield, Morehill, Airfield
**Type of living area**	Semi-urban area	Urban area	Semi-urban area	Urban area
**Economic income of the area**	Low income	High income	Low income	High income
**Annual household income of the population**	R19,601—R38,200 (mostly)	R153,801—R307,600 (mostly)	R19,601—R38,200 (mostly)	R153,801—R307,600 (mostly)
**Education of the population**	35% (secondary-level high school); 10.4% (tertiary level)	36.5% (secondary-level high school); 42.4% (tertiary level)	31.9% (secondary-level high school); 7% (tertiary level)	38.8% (secondary-level high school); 23.9% (tertiary level)
**% of the population with access to private medical aid**	Low (~9%)	High (42%)	Low (~9%)	High (37%)
**% of households with piped water inside their dwellings**	58.7%	94.1%	59.9%	79.7%
**% of households with flush toilets connected to the sewer**	85.3%	96.2%	96.3%	81.9%
**% of households with electricity for lighting**	91.9%	97.8%	91.3%	79.5%
**% of dwellings which are formal constructions**	73.8%	97.2%	86.7%	85.4%
**% of households that receive weekly refuse removal**	87.1%	94.9%	97.4%	86.5%
**Race group represented by the population**	99.2% Black African; 0.1% White; 0.3% coloured; 0.1% Indian/Asian	42% Black African; 52.5% White; 2.5% Coloured; 1.9% Indian/Asian	98.8% Black African; 0.2% White; 0.4% Coloured; 0.2% Indian/Asian	45.2% Black African; 38.1% White; 2.1% Coloured; 13.9% Indian/Asian

For the TMM, wastewater feeding into the WWTW are sourced only from sewer networks connected to homes, business and industry. So, in theory, there should be no wastewater run-off from the environment. However, illegal connections to the sewer networks do sometimes occur, so one cannot completely exclude the possibility of environmental wastewater run-off into the sewer network. For the EMM, the sewage network is a combined system, meaning that storm water and other environmental wastewater run-off can flow into the sewer network.

### Raw untreated sewage sample collection

Raw untreated sewage samples were collected from the WWTWs over the period December 2020 to November 2022, with collections occurring on the first Tuesday morning of each month. One litre volumes of sewage samples were collected at the inlet of the WWTW. Samples were packaged into cooler boxes (with ice packs) and transported (for ~2 hours) to the Centre for Enteric Diseases (CED) at the National Institute for Communicable Diseases (NICD). Upon receipt at CED, sewage samples were stored at 4°C, for up to 24 hours.

### DNA extraction from sewage samples

Within 24 hours of receipt of a sewage sample, it was processed to extract genomic DNA. Two-hundred and fifty millilitre (250 ml) of sample was processed using the QIAamp Fast DNA Stool Mini Kit (QIAGEN, Hilden, Germany) including bead beating, as per the method described by Knudsen *et al* [27]. The quantity and quality of DNA extractions were measured using a NanoDrop 1000 spectrophotometer (Thermo Scientific, Waltham, MA, USA). DNA extractions (in 1.5 ml tubes) were stored in a plastic box at a temperature of -20°C.

### Metagenomics sequencing

The box with tubes of frozen DNA samples were moved to a biosafety transport box containing dry ice, and the box was shipped to Admera Health Biopharma Services, South Plainfield, NJ, USA, for shotgun metagenomics sequencing of the DNA samples. Libraries were prepared using the KAPA HyperPrep PCR-free Kit (Roche Diagnostics Corporation, Indianapolis, IN, USA). Sequencing was performed using an Illumina NovaSeq (Illumina, San Diego, CA, USA) using paired-end (PE) 2 x 150-bp reads. We targeted 70 million reads (35 million PE reads) per sample, which resulted in ~11 Gb of raw sequencing data per sample. Quality inspection of raw reads was performed using FastQC and MultiQC [28]. Raw reads were trimmed (including adaptor removal) using BBDuk (https://jgi.doe.gov/data-and-tools/software-tools/bbtools/) with a minimum Phred quality score of 20 and minimum length of 50 bp. Trimmed reads were then used as input data for downstream analyses.

### Analysis of metagenomics sequencing data

In order to quantify the AMR genes in the metagenomes, we used the KMA tool to align reads against the ResFinder database (accessed January 2023) [29,30]. Briefly, KMA uses a seed-and-extend strategy of first mapping reads based on exact-matching k-mers and then calculates exact alignment scores for the assignment of query sequences to reference sequences. It then utilizes a “winner takes it all” approach to identify the most parsimonious sources in even highly redundant databases. For bacterial quantification, trimmed sequencing reads were aligned to the SILVA database of ribosomal RNA sequences (accessed January 2023) (https://www.arb-silva.de/), using the KMA tool [31].

### Normalization of read counts, calculation of relative abundance, and analysis of data

KMA mapstat files (produced with the “extended feature” flag) which summarize the mapping and alignment results were saved for all samples. The relative abundance for AMR was calculated as follows: the number of ResFinder-aligned sequence fragments were adjusted to gene-length (kilobases) and the number (millions) of bacterial fragments assigned in each metagenome to obtain the Fragments per Kilobase gene per Million bacterial fragments (FPKM) as previously described [32].

Relative abundance data were further analyzed and visualized using R Software version 4.3.0 and RStudio Software version 2023.06.1+524 (https://posit.co/download/rstudio-desktop/). Statistical analysis of data was performed using the Wilcoxon rank-sum test, including a p-value cutoff of 0.05.

Alignment counts to the ResFinder database, were further analyzed using DESeq2 [33], to test for differential abundance of AMR genes between sites. The following DESeq2 parameters were used: estimateSizeFactors with type set to “poscounts” and the Wald test was used with fitType set to “parametric”. We used an adjusted p value (q-value) of 0.1 to determine significant abundance between sites during different seasons. Volcano plots were used to visualize differential abundance analysis results.

## Results

The number of reads per sequenced sample was ~70 million reads (range: 65–75 million). On average, 0.05% of the reads per sample aligned to AMR genes at the ResFinder database, while 0.25% of the reads per sample aligned to bacterial 16S rRNA genes at the SILVA database. In total, there were alignments to 1523 AMR genes and 454 bacterial families.

**Fig 1:**
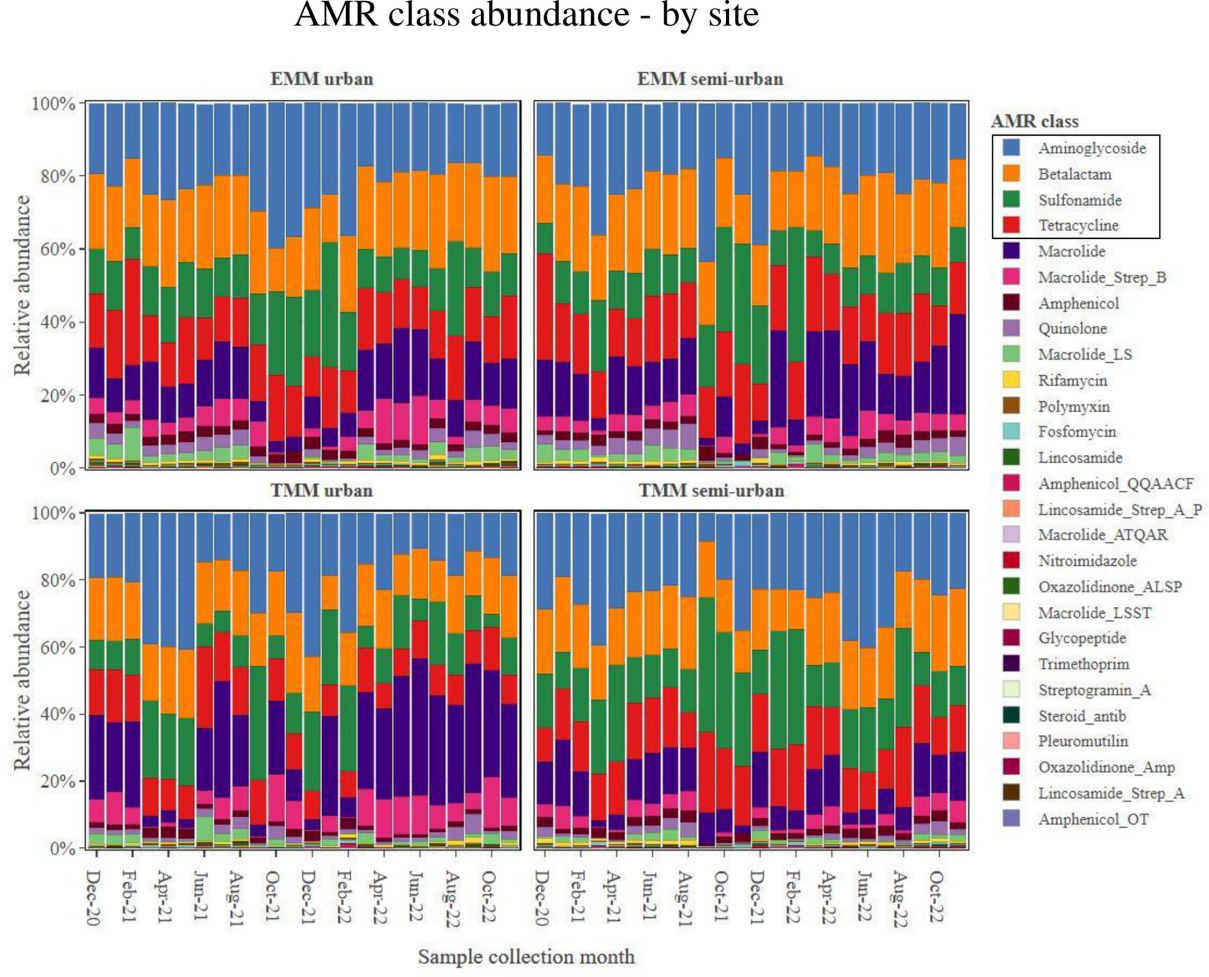
AMR class abundance, showing abundance data from samples sourced at each WWTW, by month of sampling.

**Fig 2:**
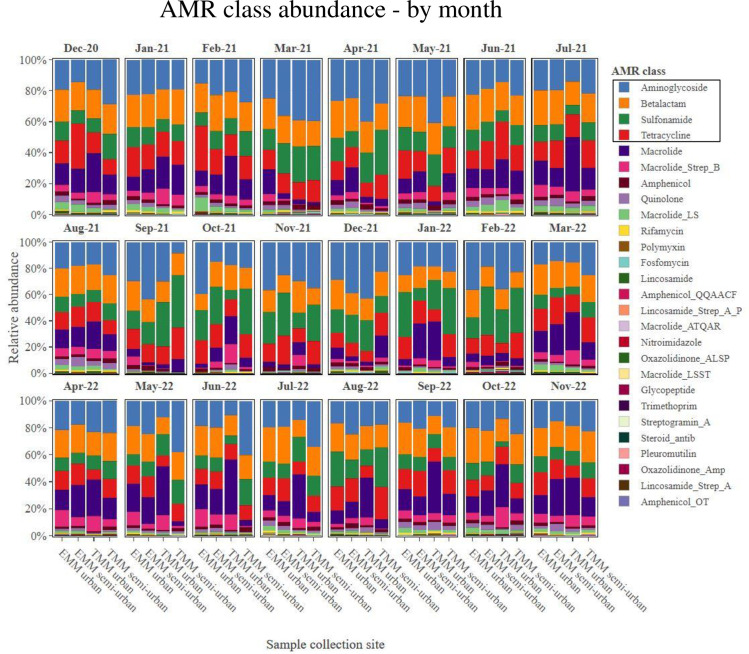
AMR class abundance, showing abundance data by month of sampling, from samples sourced at each WWTW.

**Fig 3:**
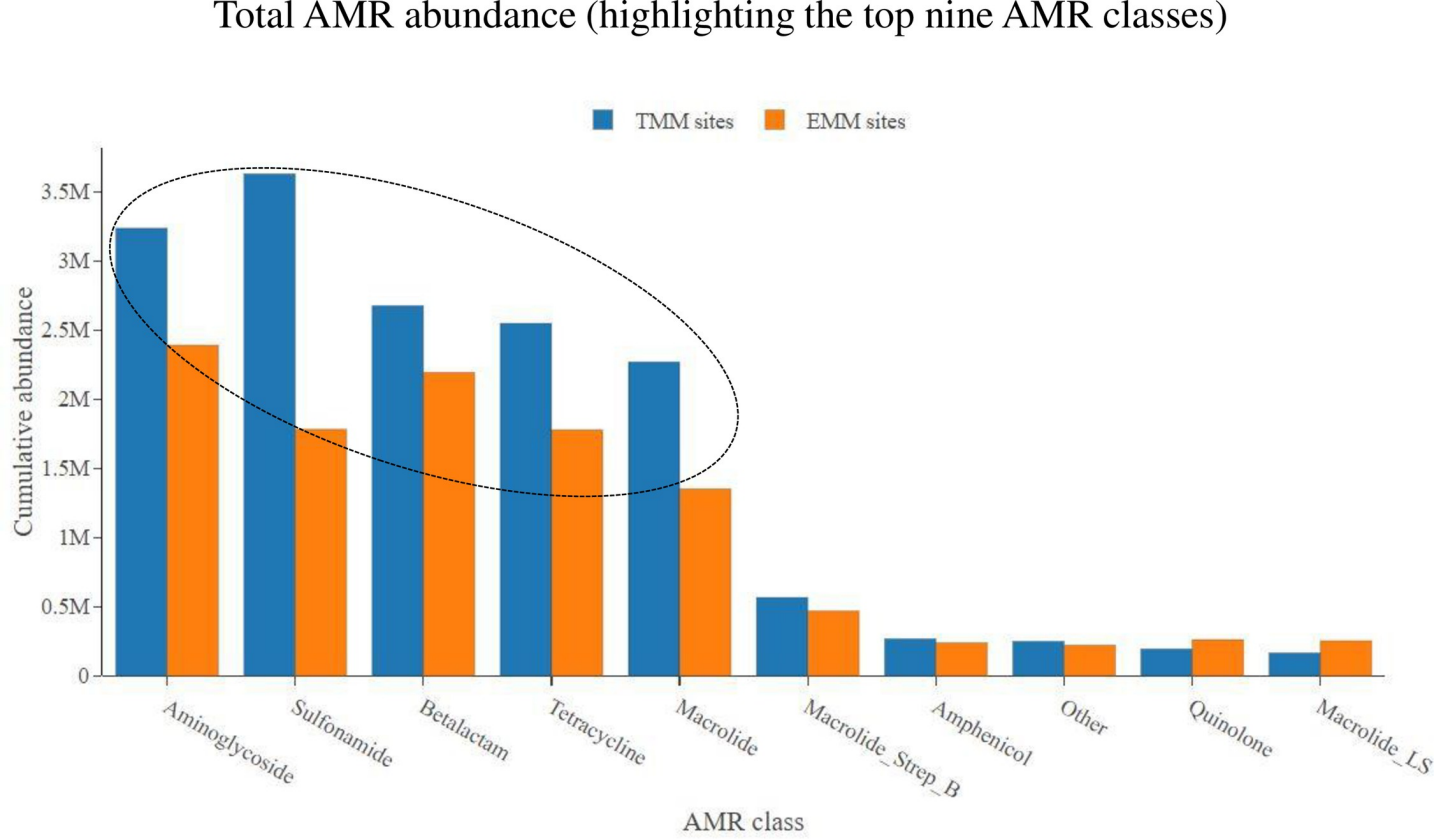
Total AMR class abundance, showing abundance data for the TMM WWTWs as compared to the EMM WWTWs. Only the top nine most abundant AMR classes are shown.

On average, collected sewage samples showed the most abundance for AMR genes belonging to the following four classes: aminoglycosides, beta-lactams, sulfonamides and tetracyclines. This AMR class trend was consistent at all study sites over the 24-month study period (Figs 1 and 2). Stratifying by metropolitan municipality, the TMM showed an overall higher abundance of AMR as compared to the EMM (p = 0.035). This was particularly notable when looking at the most abundant AMR classes, in particular the aminoglycosides, sulfonamides, beta-lactams, tetracyclines and macrolides (Fig 3). Looking at these most abundant AMR classes, the aminoglycosides (p = 0.006), sulfonamides (p = 0.019) and macrolides (p = 0.047) showed overall higher abundance at the TMM as compared to EMM (with statistically significant differences); while for beta-lactams (p = 0.863) and tetracyclines (p = 0.945), the overall higher abundance at the TMM as compared to EMM was not statistically significant (Fig 3).

**Fig 4:**
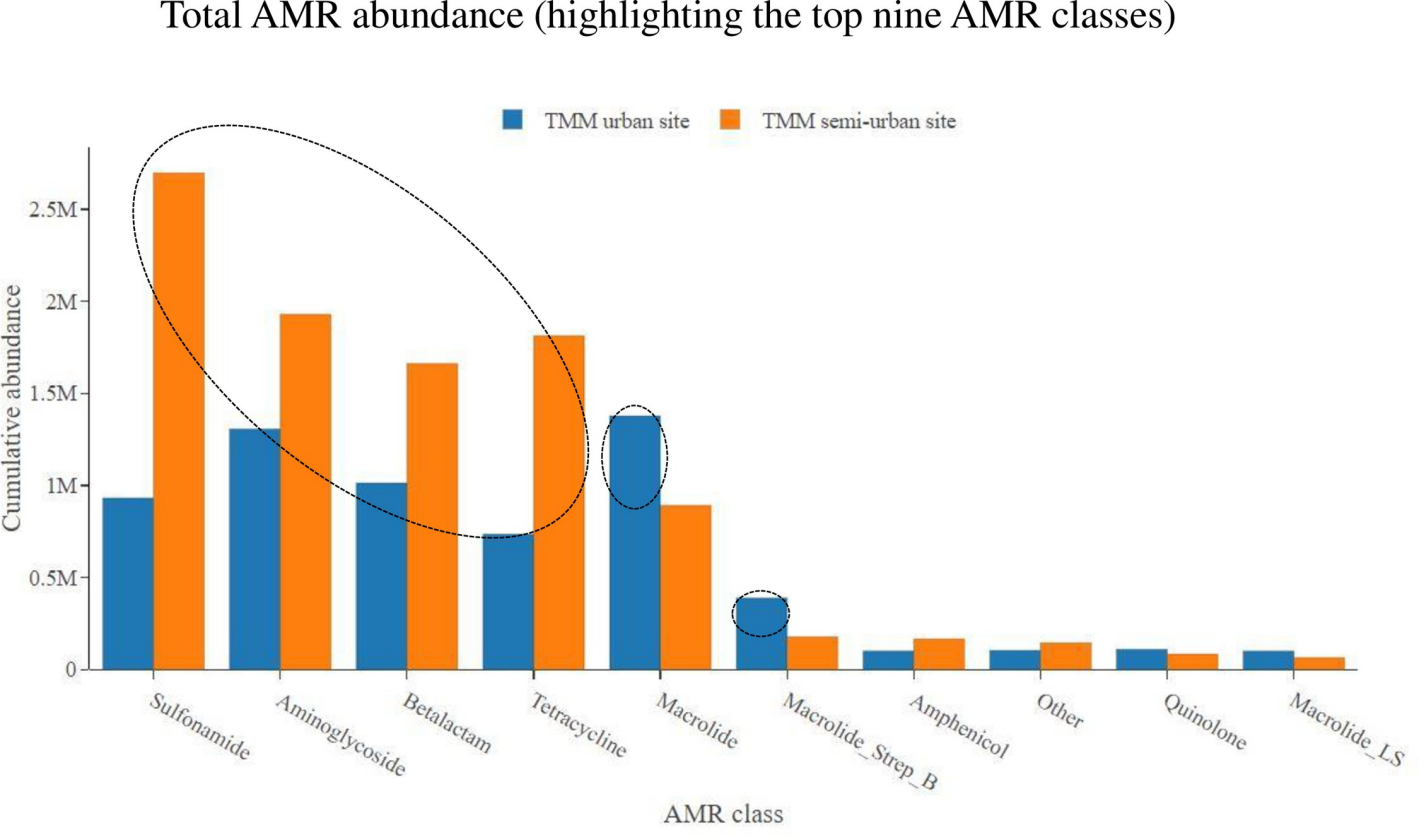
Total AMR class abundance, showing abundance data for the TMM urban WWTW as compared to the TMM semi-urban WWTW. Only the top nine most abundant AMR classes are shown.

**Fig 5:**
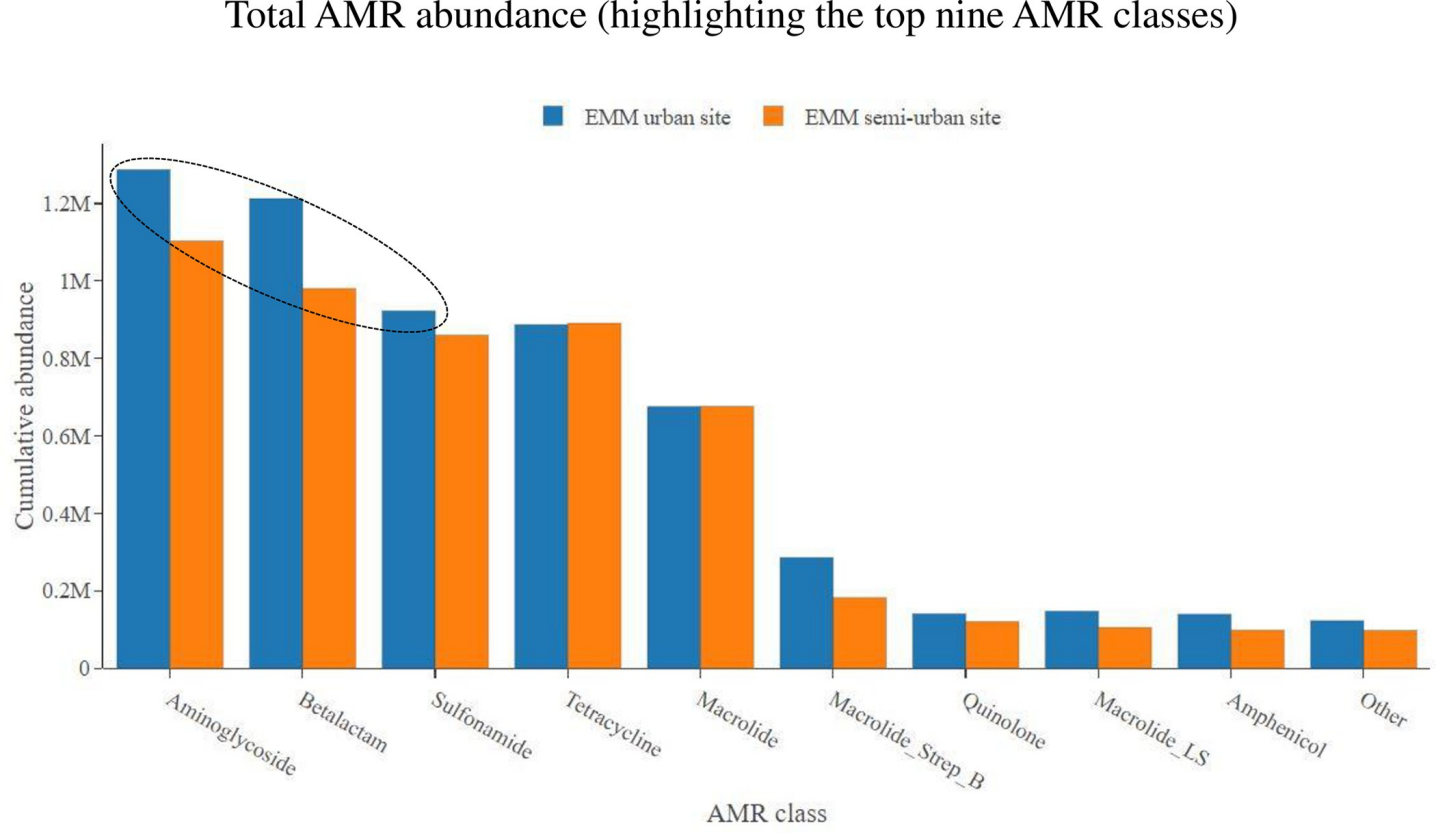
Total AMR class abundance, showing abundance data for the EMM urban WWTW as compared to the EMM semi-urban WWTW. Only the top nine most abundant AMR classes are shown.

Stratifying within metropolitan municipality, comparing WWTWs which serve semi-urban low-income areas with WWTWs which serve urban high income areas, we showed the following results. Over the 24-month study period, no statistically significant difference was noted for total AMR abundance at the TMM semi-urban WWTW as compared to the TMM urban WWTW (p = 0.797). However, looking at the most abundant AMR classes, the TMM semi-urban WWTW showed a higher abundance of aminoglycosides (p = 0.007) as compared to the TMM urban WWTW (Fig 4). In contrast, there was a higher abundance of macrolides (p = 0.016) and macrolides_streptogramin B (p = 0.006) at the TMM urban WWTW as compared to TMM semi-urban WWTW. No statistically significant differences in abundance were noted for sulfonamides (p = 0.056), beta-lactams (p = 0.059) and tetracyclines (p = 0.062) (Fig 4). The situation looked somewhat different at the second municipality (EMM). The EMM urban WWTW showed an overall higher total abundance of AMR as compared to the EMM semi-urban WWTW (p = 0.040). Looking at the most abundant AMR classes, the EMM urban WWTW showed a higher abundance of aminoglycosides (p < 0.001), beta-lactams (p = 0.044) and sulfonamides (p = 0.005) as compared to the EMM semi-urban WWTW (Fig 5). For other AMR classes, including tetracyclines (p = 0.489) and macrolides (p = 0.975), no statistically significant differences in abundance were noted (Fig 5).

**Fig 6:**
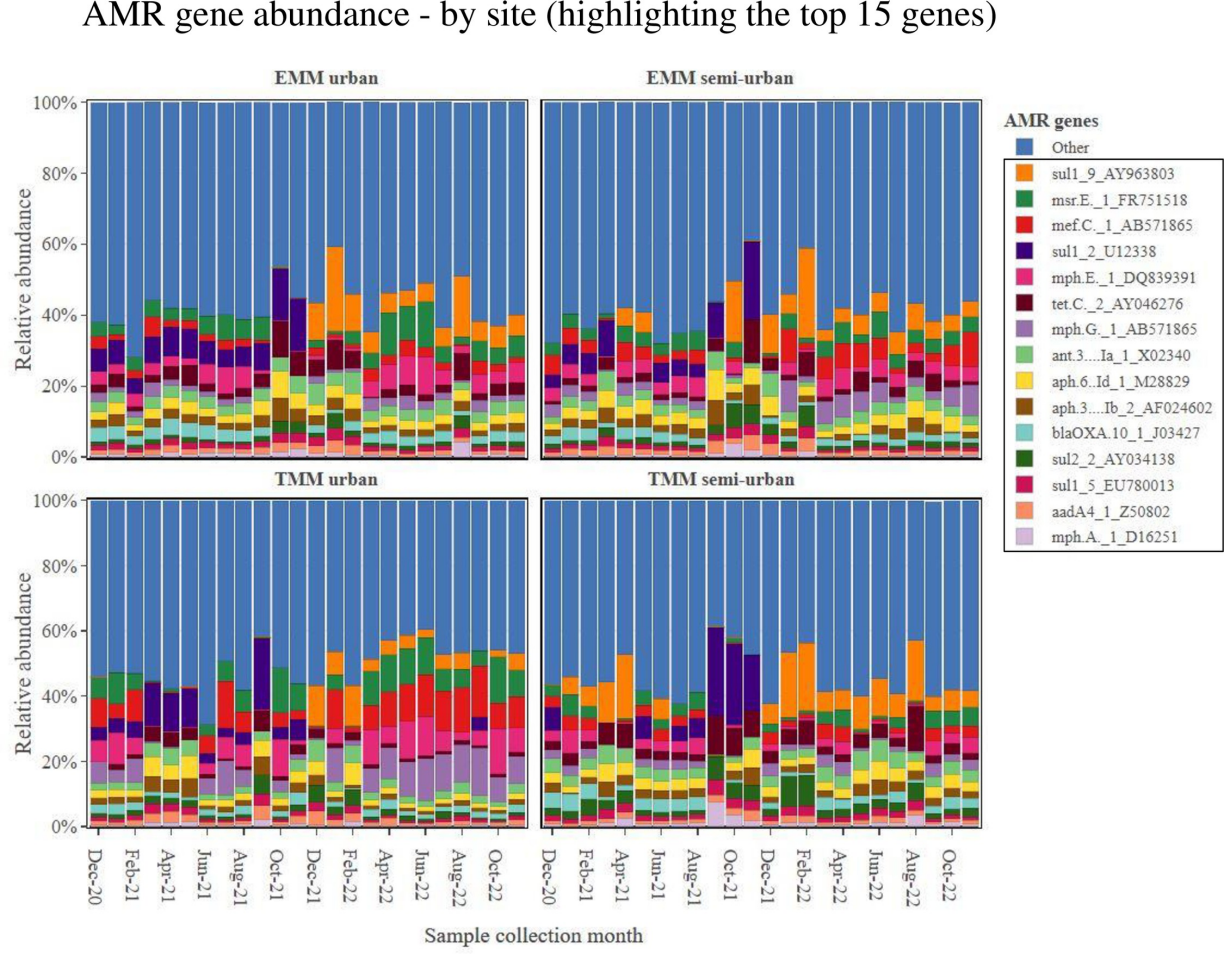
AMR gene abundance, showing abundance data from samples sourced at each WWTW, by month of sampling. The top 15 most abundant AMR genes are shown, while the remainder of the genes are grouped together and shown as ‘Other’.

**Fig 7:**
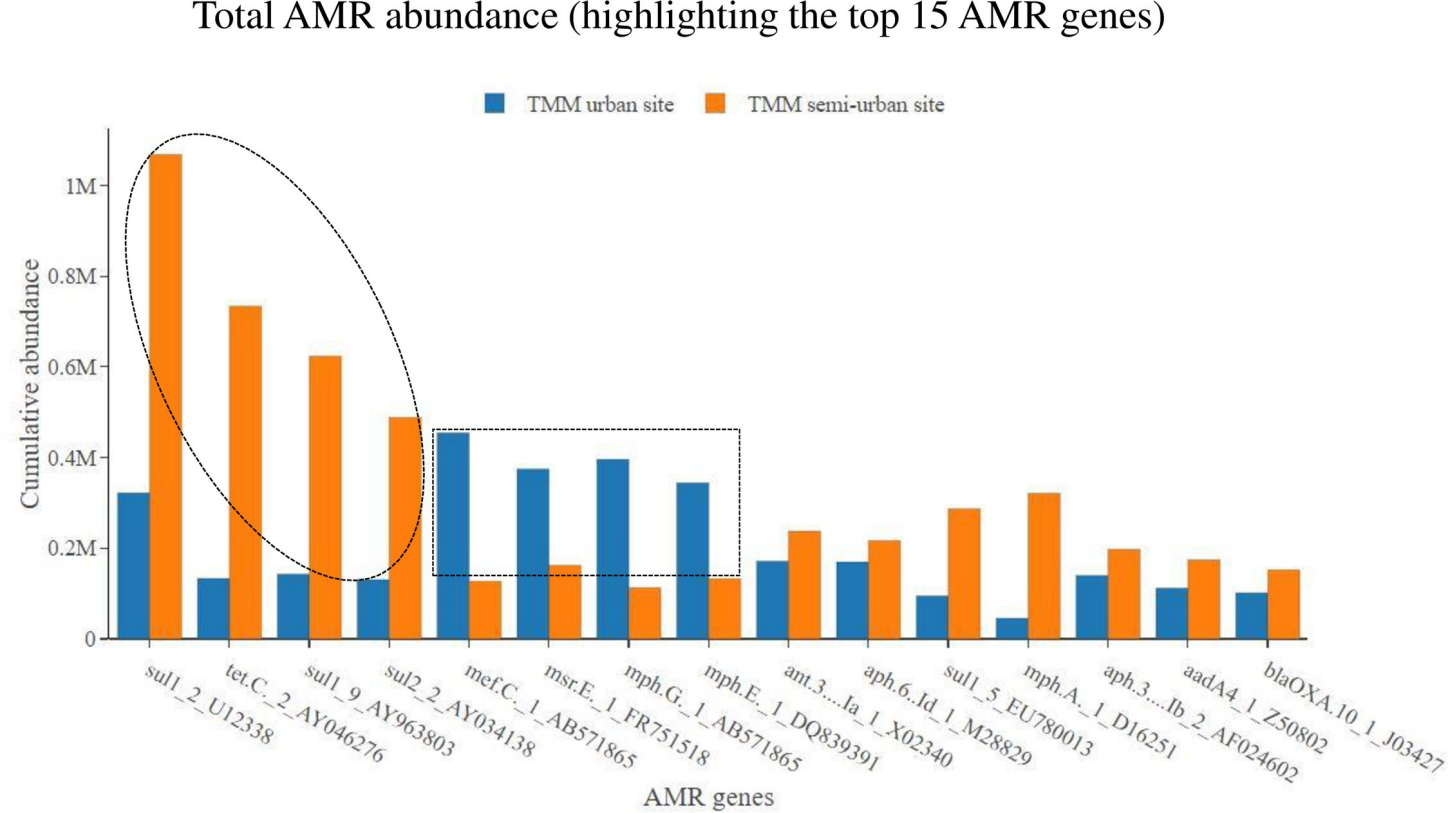
Total AMR gene abundance, showing abundance data for the TMM urban WWTW as compared to the TMM semi-urban WWTW. Only the top 15 most abundant AMR genes are shown.

**Fig 8:**
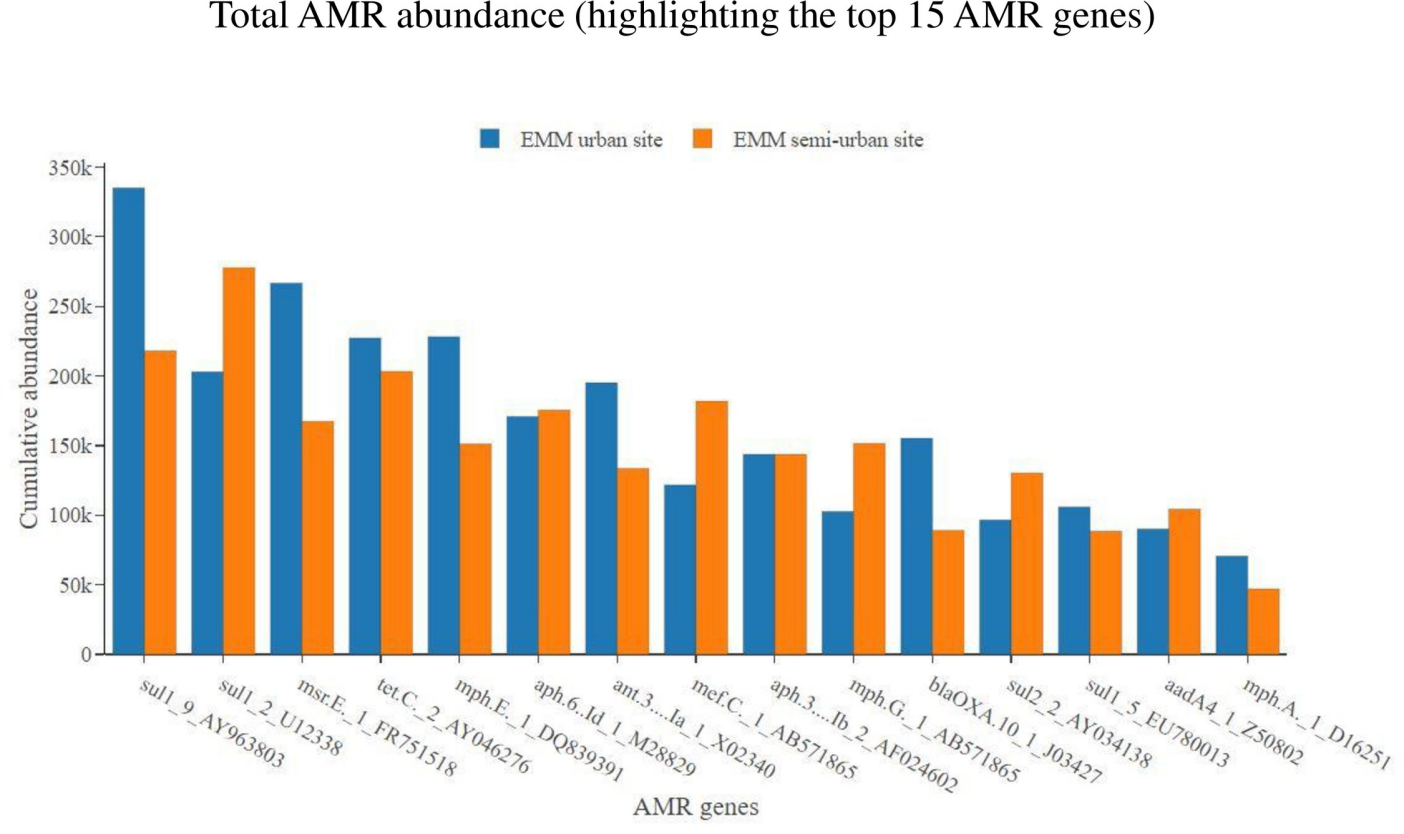
Total AMR gene abundance, showing abundance data for the EMM urban WWTW as compared to the EMM semi-urban WWTW. Only the top 15 most abundant AMR genes are shown.

At the AMR gene level, on average, samples showed the most abundance for the *sul1* gene (encoding sulfonamide resistance)—thereafter, the next most abundant 14 AMR genes included a range of genes encoding resistance to sulfonamides, aminoglycosides, macrolides, tetracyclines and beta-lactams (Fig 6). These 15 most abundant AMR genes accounted for 48% of all AMR genes detected in the collected samples (out of a total AMR gene count of 1523) (Fig 6). Stratifying within metropolitan municipality, comparing WWTWs which serve semi-urban low-income areas with WWTWs which serve urban high-income areas, we showed the following results. For some AMR genes (*sul1*, *sul2* and *tetC*); the TMM semi-urban WWTW showed an overall higher abundance of AMR as compared to the TMM urban WWTW [*sul1*-AY963803 (p = 0.005), *sul2*-AY034138 (p <0.001) and *tetC*-AY046276 (p <0.001)] (Fig 7). However, for other AMR genes (*mefC*, *msrE*, *mphG* and *mphE*); the TMM urban WWTW showed an overall higher abundance of AMR as compared to the TMM semi-urban WWTW [*mefC*-AB571865 (p = 0.003), *msrE*-FR751518 (p = 0.005), *mphG*-AB571865 (p = 0.003) and *mphE*-DQ839391 (p = 0.016)] (Fig 7). Once again, the situation looked somewhat different at the second municipality the EMM. For the EMM semi-urban WWTW and EMM urban WWTW, overall AMR gene abundance data (for the 15 most abundant AMR genes) were mostly homogeneous at the two WWTW (Fig 8). As an example, no statistically significant differences in AMR gene abundance data was noted for *sul1*-AY963803 (p = 0.261), *sul1*-U12338 (p = 0.088) and *mphE*-DQ839391 (p = 0.088). Notable exceptions included *msrE*-FR751518 (p = 0.049) and *tetC*-AY046276 (p < 0.001) that showed some higher abundance at the EMM urban WWTW as compared to the EMM semi-urban WWTW (Fig 8).

**Fig 9:**
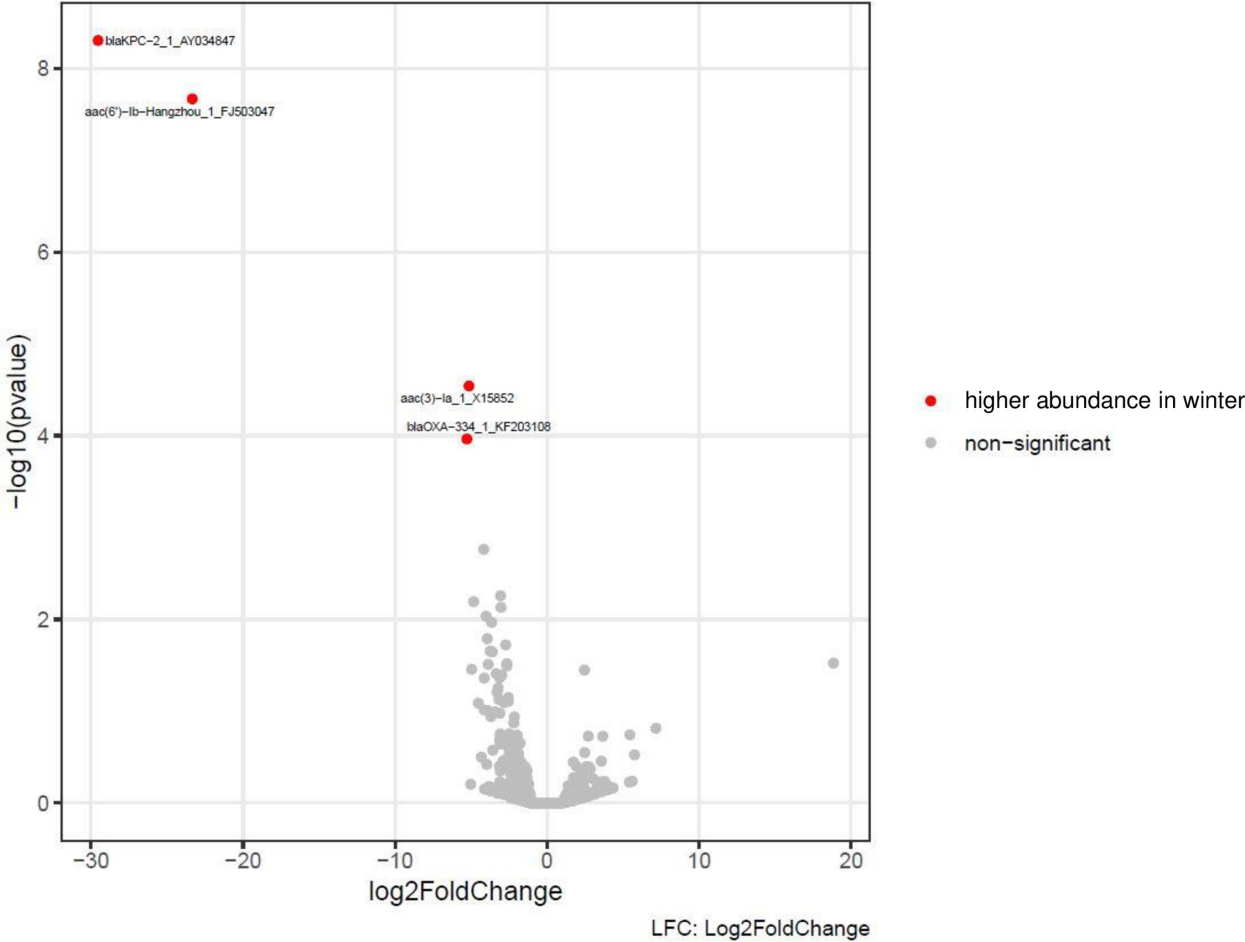
Differential abundance data analysis of AMR genes from samples sourced at the TMM semi-urban WWTW. Data were compared following stratification by season, here comparing data between the summer and winter periods.

Finally, a differential abundance analysis of the AMR genes was performed. For each individual WWTW, data were compared between seasons. The TMM WWTWs showed some notable variance in AMR gene abundance between seasons, while the EMM WWTWs showed little variance (Table 2). In total, significant seasonal abundance differences were found for only six AMR genes, which included: *bla*_*KPC-2*_ beta-lactamase gene (AY034847), *bla*_*KPC-34*_ beta-lactamase gene (KU985429), *bla*_*OXA-334*_ beta-lactamase gene (KF203108), all encoding resistance to carbapenems; *bla*_*NPS-1*_ beta-lactamase gene (AY027589) encoding resistance to beta-lactams; *aac(6’)-Ib-Hangzhou* gene (FJ503047), *aac(3)-Ia* gene (X15852), both encoding resistance to aminoglycosides (Table 2, Fig 9). Among these six AMR genes, the *bla*_*KPC-2*_ and *bla*_*KPC-34*_ genes showed the highest prevalence of seasonal abundance differences when comparing data within a WWTW.

**Table 2:** Differential abundance data analysis of AMR genes from samples sourced at WWTWs. **Data were compared following stratification by season.** Summer (included the months of December, January, February), autumn (included the months of March, April, May), winter (included the months of June, July, August) and spring (included the months of September, October, November).

	TMM semi-urban WWTW	TMM urban WWTW	EMM semi-urban WWTW	EMM urban WWTW
**Summer compared to Winter**	*bla*_*KPC-2*_ gene (AY034847) more abundant in Winter; *bla*_*OXA-334*_ gene (KF203108) more abundant in Winter; *aac(3)-Ia gene* (X15852) more abundant in Winter; *aac(6’)-Ib-Hangzhou* gene (FJ503047) more abundant in Winter	No significant abundance difference in AMR genes	No significant abundance difference in AMR genes	No significant abundance difference in AMR genes
**Spring compared to Winter**	No significant abundance difference in AMR genes	No significant abundance difference in AMR genes	*bla*_*NPS-1*_ gene (AY027589) more abundant in Winter	No significant abundance difference in AMR genes
**Spring compared to Autumn**	*bla*_*KPC-2*_ gene (AY034847) more abundant in Spring	*bla*_*KPC-2*_ gene (AY034847) more abundant in Autumn; *bla*_*KPC-34*_ gene (KU985429) more abundant in Spring	No significant abundance difference in AMR genes	No significant abundance difference in AMR genes
**Autumn compared to Winter**	*bla*_*KPC-2*_ gene (AY034847) more abundant in Winter	*bla*_*KPC-2*_ gene (AY034847) more abundant in Autumn; *bla*_*KPC-34*_ gene (KU985429) more abundant in Winter	No significant abundance difference in AMR genes	No significant abundance difference in AMR genes
**Summer compared to Autumn**	*aac(6’)-Ib-Hangzhou* gene (FJ503047) more abundant in Autumn	*bla*_*KPC-2*_ gene (AY034847) more abundant in Autumn; *bla*_*KPC-34*_ gene (KU985429) more abundant in Summer	No significant abundance difference in AMR genes	No significant abundance difference in AMR genes
**Summer compared to Spring**	*bla*_*KPC-2*_ gene (AY034847) more abundant in Spring; *aac(6’)-Ib-Hangzhou* gene (FJ503047) more abundant in Spring	No significant abundance difference in AMR genes	No significant abundance difference in AMR genes	No significant abundance difference in AMR genes

## Discussion

Sewage samples showed the most abundance for AMR genes belonging to the following four classes: aminoglycosides, beta-lactams, sulfonamides and tetracyclines. This AMR class trend was consistent at all study sites over the 24-month study period. These results are in agreement with a global study of urban sewage which found that for the Africa/South America/Asia regions, the highest abundance of AMR genes were associated with tetracyclines, aminoglycosides, beta-lactams, sulfonamides and trimethoprim [15]. Stratifying by metropolitan municipality, the TMM showed an overall higher abundance of AMR as compared to the EMM.

The high levels of sulfonamide AMR gene abundance observed were not surprising, as it correlates with the extensive usage of co-trimoxazole which forms part of therapy for HIV-positive patients in South Africa’s huge antiretroviral program [34–36]. Also, sulfonamides are widely used to treat a wide variety of bacterial infections and some fungal infections, and in particular urinary tract infections. High levels of beta-lactam AMR gene abundance observed correlates well with the beta-lactams penicillin, amoxicillin and amoxicillin/clavulanic acid being the leading prescribed antimicrobial class in South Africa. Also, globally, beta-lactams are the most widely prescribed antimicrobial and is used to treat a wide variety of bacterial infections [37]. High levels of aminoglycoside AMR gene abundance observed could be ascribed to the recent global resurrection and resurgence in aminoglycoside use for the treatment of mostly Gram-negative bacterial infections (and some Gram-positives) in humans and animals. Aminoglycosides particularly combine well and show good synergy with other antimicrobials, particularly with the beta-lactams, and this provides expanded effectiveness for the treatment of multidrug-resistant organisms [38–40]. High levels of tetracycline AMR gene abundance correlates with an antimicrobial class that is frequently employed as a therapeutic option in human and animal healthcare due to their broad spectrum of activity, as well as their low cost as compared to other antimicrobials. In addition to therapeutic purposes, tetracyclines are among the most common antimicrobials used as growth promoters in the practice of farming food-producing animals (livestock, poultry, etc.) [41,42].

Stratifying within metropolitan municipality, comparing WWTWs which serve semi-urban low-income areas with WWTWs which serve urban high income areas, we noticed some differences in total AMR class abundance data. For the AMR classes macrolides and macrolides_streptogramin B; the TMM urban WWTW showed an overall higher abundance of AMR genes as compared to the TMM semi-urban WWTW. This was not surprising, as urban areas are more associated with private healthcare, where the more expensive antimicrobials (like macrolides) are prescribed [37,43,44]. Affluent persons living in urban high-income areas often expect and even demand prescription of antimicrobials, even if not required (like for viral infections); they would even demand the most expensive antimicrobials available (like macrolides), thinking that more expensive must be better. Medical practitioners in private healthcare are often pressured into meeting the demands of patients, for fear of patients leaving their practice to seek unnecessary medication elsewhere [43,45]. Contrary, for the AMR class aminoglycosides, the TMM semi-urban WWTW showed an overall higher abundance of AMR genes as compared to the TMM urban WWTW. This would align with semi-urban areas which are associated with public healthcare, where prescription is driven by the more affordable antimicrobials, like the aminoglycosides [37,46]. Interestingly, as previously mentioned, the situation looked somewhat different at the second metropolitan municipality (EMM). The EMM urban WWTW showed an overall higher abundance for some of the predominant AMR classes (including aminoglycosides, beta-lactams and sulfonamides), as compared to the EMM semi-urban WWTW. For other AMR classes (including tetracyclines and macrolides), no significant differences in abundance were noted.

The high prevalence of sulfonamide resistance genes (*sul1* and *sul2*) was not surprising. In particular, the *sul1* gene has previously been reported to show a high prevalence in wastewater samples from African countries [15,22,25]. Following the *sul* genes, the *tetC* gene (encoding tetracycline resistance) was the next most abundant AMR gene detected among all samples. AMR genes associated with macrolide resistance also made up a large proportion of the top 15 genes, including *msrE*, *mefC*, *mphE*, *mphG* and *mphA*. AMR genes associated with aminoglycoside resistance also made up a large proportion of the top 15 genes, including *ant(3’’)-Ia*, *aph(6)-Id*, *aph(3’’)-Ib* and *aadA4*. Lastly, a *bla*_OXA-10_ gene (associated with beta-lactam resistance) encoding an extended-spectrum beta-lactamase (ESBL), completed the top 15 AMR genes. Among all AMR genes associated with beta-lactam resistance, we found that the gene which recorded the highest number of read mapping (most detected) was a *bla*_OXA_ gene (*bla*_OXA-10_). This was somewhat surprising, since globally among ESBL gene classes/variants, the most prevalent and most described are *bla*_TEM_, *bla*_SHV_, and *bla*_CTX-M_ [47,48]. Of concern with our finding that a *bla*_OXA_ gene is well detected, is that *bla*_OXA_ gene variants encode resistance to carbapenems, an antimicrobial considered to be among one of the last options for patient treatment. Stratifying within metropolitan municipality, for the TMM, AMR gene abundance data mostly reflected what was observed at the AMR class level. For some AMR genes associated with resistance to macrolides (*mefC*, *msrE*, *mphG* and *mphE*), samples from the TMM urban WWTW showed an overall higher abundance of the AMR genes as compared to the TMM semi-urban WWTW. Contrary, for some AMR genes associated with resistance to sulfonamides and tetracyclines (*sul1*, *sul2* and *tetC*), samples from the TMM semi-urban WWTW showed an overall higher abundance of the AMR genes as compared to the TMM urban WWTW. At the EMM, AMR gene abundance data also reflected what was observed at the AMR class level. For samples from the EMM semi-urban WWTW and EMM urban WWTW, overall AMR gene abundance data (for the 15 most abundant AMR genes) were mostly homogeneous at the two WWTW.

Our final analysis of data included the following. Data from alignment counts were further analyzed using DESeq2 to perform a differential abundance analysis of the AMR genes. Data from WWTWs were compared following stratification by season. Summer (included the months of December, January, February), autumn (included the months of March, April, May), winter (included the months of June, July, August) and spring (included the months of September, October, November). For each individual WWTW, data were compared between seasons. The TMM WWTWs showed some notable variance in AMR gene abundance between seasons, while the EMM WWTWs showed little variance. In total, significant seasonal abundance differences were found for only six AMR genes, which included: *bla*_*KPC-2*_, *bla*_*KPC-34*_, *bla*_*OXA-334*_, *bla*_*NPS-1*_, *aac(6’)-Ib-Hangzhou*, and *aac(3)-Ia*. Among these six AMR genes, the *bla*_*KPC-2*_ and *bla*_*KPC-34*_ beta-lactamase genes showed the highest prevalence of seasonal abundance differences when comparing data within a WWTW. The general trend was to see higher abundances of AMR genes in colder seasons, when comparing seasonal data within a WWTW, although there were a few exceptions.

Limitations of our study included the following. Our study should be considered a pilot investigating the feasibility of a metagenomics approach to investigate AMR gene presence in wastewater in South Africa. Due to the relatively high cost of metagenomics analysis, our study could only include a limited number of sewage samples, that of “once a month” grab samples from WTWWs. For future analysis, it would be best to analyze composite sewage samples taken daily over an extended period of time. Also, our limited “once a month” grab sampling does not provide enough data to answer questions related to abundance of AMR genes versus antimicrobial consumption data. To try and make associations between abundance of AMR genes and antimicrobial consumption data, a more comprehensive sampling strategy would be required. Then, even with a more comprehensive sampling strategy, including composite samples taken daily over an extended time period, it will still remain challenging to attempt linking abundance of AMR genes to antimicrobial consumption data, because there are limited stratified municipal/regional antimicrobial consumption data available. Neither the clinical, agricultural, or veterinary sectors are sharing or publishing much of these municipal/regional data.

## Conclusions

Our 24-month study used metagenomics to investigate AMR abundance in raw sewage from WWTWs in two metropolitan municipalities in the Gauteng Province of South Africa. We showed a diversity of AMR abundance data in samples from one WWTW as compared to another WWTW, which highlights the complexities of the AMR ecology. These complexities are known to include multiple drivers of AMR which include selective pressures associated with antimicrobial use/abuse and factors (like social and economic factors) which affect the spread of antimicrobial-resistant microorganisms [49,50]. To some extent, we showed that differences in AMR abundance data could be aligned with the socioeconomic status of the population served by the WWTW, as seen for macrolide resistance which showed the highest abundance in samples from a WWTW serving an urban high-income area. To some extent, we showed that differences in AMR gene abundance data could be aligned with season, where the general trend was to see higher abundances of some AMR genes in colder seasons, when comparing seasonal data within a WWTW.

Our study investigated wastewater samples in only one province of South Africa, from WWTWs located within close proximity to one another. We would require a more widespread investigation at WWTWs distributed across all regions/provinces of South Africa, in order to describe a more comprehensive profile of AMR abundance across the country. Globally, wastewater surveillance programs are rapidly expanding to elucidate the burden and spread of multiple pathogens and associated characteristics. The NICD, South Africa already has a countrywide wastewater surveillance program in place which primarily investigates for Polio and COVID. It would be worthwhile to see this NICD program expand to see the inclusion of surveillance for AMR.
